# Tri­benzyl­ammonium chloride

**DOI:** 10.1107/S1600536814009246

**Published:** 2014-04-30

**Authors:** Waly Diallo, Libasse Diop, Laurent Plasseraud, Hélène Cattey

**Affiliations:** a Laboratoire de Chimie Minérale et Analytique (LACHIMIA), Département de Chimie, Faculté des Sciences et Techniques, Université Cheikh Anta Diop, Dakar, Senegal; b ICMUB UMR 6302, Université de Bourgogne, Faculté des Sciences, 9 avenue Alain Savary, 21000 Dijon, France

## Abstract

Single crystals of the title salt, C_21_H_21_NH^+^·Cl^−^, were isolated as a side product from the reaction involving [(C_6_H_5_CH_2_)_3_NH]_2_[HPO_4_] and Sn(CH_3_)_3_Cl in ethanol. Both the cation and the anion are situated on a threefold rotation axis. The central N atom in the cation has a slightly distorted tetra­hedral environment, with angles ranging from 107.7 to 111.16 (10)°. In the crystal, the tri­benzyl­ammonium cations and chloride anions are linked through N—H⋯Cl and C—H⋯Cl hydrogen bonds, leading to the formation of infinite chains along [001]. The crystal studied was a merohedral twin.

## Related literature   

For related crystal structures containing the tri­benzyl­ammonium cation, see: Kozhomuratova *et al.* (2007 ▶); Jarvinen *et al.* (1988 ▶); Guo *et al.* (2010 ▶); Zeng *et al.* (1994 ▶); Faza­eli *et al.* (2010 ▶); Guan *et al.* (2013 ▶); Yousefi *et al.* (2007 ▶); Gueye *et al.* (2012 ▶); Traore *et al.* (2013 ▶). For details of the treatment of intensity data from a twinned crystal, see: Spek (2009 ▶).
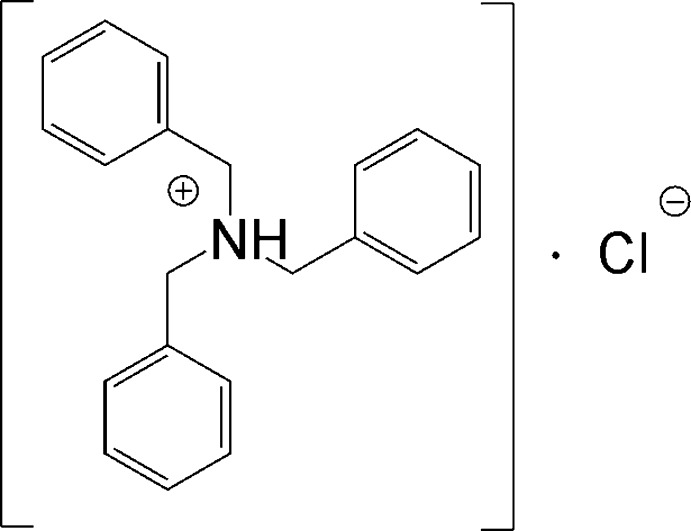


## Experimental   

### 

#### Crystal data  

C_21_H_22_N^+^·Cl^−^
*M* *_r_* = 323.85
Trigonal, 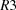

*a* = 15.3833 (8) Å
*c* = 6.7051 (3) Å
*V* = 1374.15 (18) Å^3^
*Z* = 3
Mo *K*α radiation
μ = 0.21 mm^−1^
*T* = 115 K
0.47 × 0.27 × 0.12 mm

#### Data collection  

Nonius KappaCCD diffractometer
Absorption correction: multi-scan (Blessing, 1995 ▶) *T*_min_ = 0.923, *T*_max_ = 0.963
1884 measured reflections
1047 independent reflections
1045 reflections with *I* > 2σ(*I*)
*R*_int_ = 0.016

#### Refinement  

*R*[*F* ^2^ > 2σ(*F* ^2^)] = 0.021
*wR*(*F* ^2^) = 0.052
*S* = 1.10
1047 reflections
71 parameters
1 restraint
H-atom parameters constrained
Δρ_max_ = 0.12 e Å^−3^
Δρ_min_ = −0.11 e Å^−3^
Absolute structure: Flack parameter determined using 348 quotients [(*I* ^+^)−(*I* ^−^)]/[(*I* ^+^)+(*I* ^−^)] (Parsons *et al.*, 2012 ▶)
Absolute structure parameter: 0.01 (4)

### 

Data collection: *COLLECT* (Nonius, 1998 ▶); cell refinement: *DENZO-SMN* (Otwinowski & Minor, 1997 ▶); data reduction: *DENZO-SMN*; program(s) used to solve structure: *SIR92* (Altomare *et al.*, 1993 ▶); program(s) used to refine structure: *SHELXL97* (Sheldrick, 2008 ▶); molecular graphics: *ORTEP-3 for Windows* (Farrugia, 2012 ▶) and *Mercury* (Macrae *et al.*, 2008 ▶); software used to prepare material for publication: *WinGX* (Farrugia, 2012 ▶).

## Supplementary Material

Crystal structure: contains datablock(s) global, I. DOI: 10.1107/S1600536814009246/wm5019sup1.cif

Structure factors: contains datablock(s) I. DOI: 10.1107/S1600536814009246/wm5019Isup2.hkl

Supporting information file. DOI: 10.1107/S1600536814009246/wm5019Isup3.cml

CCDC reference: 999186

Additional supporting information:  crystallographic information; 3D view; checkCIF report

## Figures and Tables

**Table 1:** Hydrogen-bond geometry (Å, °)

*D*—H⋯*A*	*D*—H	H⋯*A*	*D*⋯*A*	*D*—H⋯*A*
N—H⋯Cl^i^	1.00	2.00	3.004 (2)	180
C1—H1*B*⋯Cl	0.99	2.70	3.5470 (18)	144
C3—H3⋯Cl^i^	0.95	3.06	3.683 (2)	125

Symmetry code: (i) 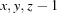.

**Fig. 1.**
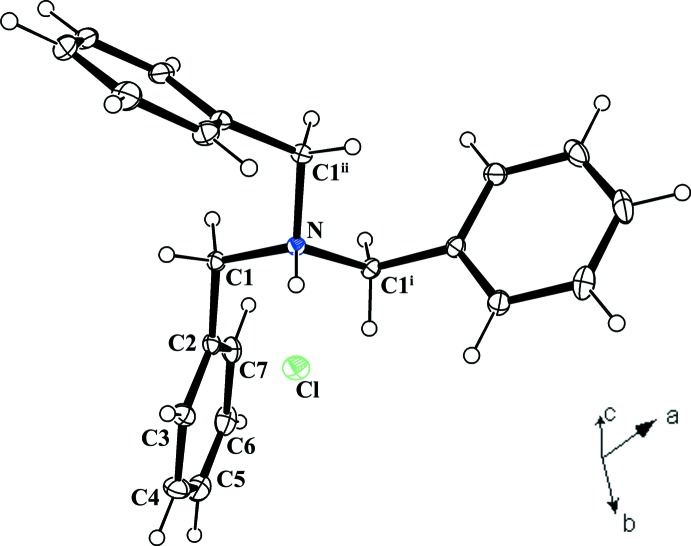
The molecular structure of the title compound with atom labeling. Displacement ellipsoids are draw at the 30% probability level. [Symmetry codes: (i) -x + y+1, -x + 1, z; (ii) -y + 1, x-y, z.]

**Fig. 2.**
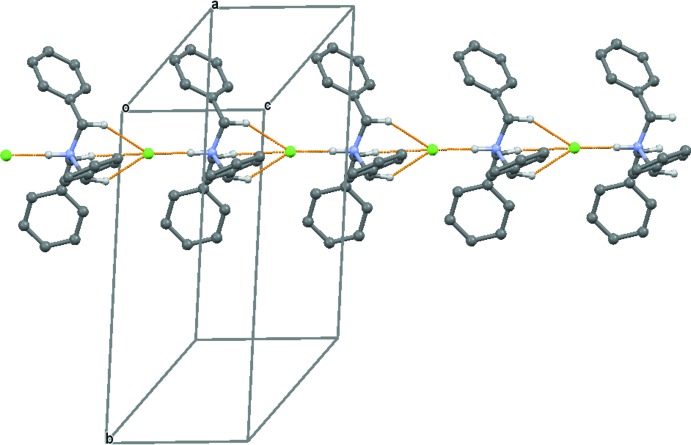
The crystal packing of the title compound showing a chain-like arrangement along [001] through N—H···Cl and C—H···Cl interactions (dashed orange lines; H atoms not involved in hydrogen bonding were omitted for clarity). Colour code: C dark grey, H light grey, N blue, Cl green.

## supplementary crystallographic information

### 1. Comment

Tribenzylammonium cations are often used to stabilize metal complex-anions such as [(C_6_H_5_CH_2_)_3_NH·C_6_H_5_CH_2_NH_2_][CuCl_4_] (Zeng *et al.*, 1994), (Bz_3_NH)_3_[Mo_6_OCl_13_] and (Bz_3_NH)_2_[Mo_6_Cl_14_]·2CH_3_CN (Bz is benzyl; Kozhomuratova *et al.*, 2007), [(C_6_H_5_CH_2_)_3_NH][AuCl_4_] (Fazaeli *et al.*, 2010), 2[C_21_H_22_N^+^]·[*M*Cl_6_]^2-^ (*M* = Se, Re, Te) (Guo *et al.*, 2010), 2[C_21_H_22_N^+^]·[CoCl_4_]^2-^ and 2[C_21_H_22_N^+^]·[CuCl_4_]^2-^ (Guan *et al.*, 2013). In the course of our ongoing studies on organotin(IV) chemistry, we serendipitously isolated the title salt, tribenzylammonium chloride C_21_H_21_NH^+^·Cl^-^, from the reaction of [(C_6_H_5_CH_2_)_3_NH]_2_[HPO_4_] with Sn(CH_3_)_3_Cl. Together with C_21_H_21_NH^+^·Cl^-^, we suggest the formation of the tin(IV) compound, [(C_6_H_5_CH_2_)_3_NH][HPO_4_SnMe_3_]. Howver, we were not successful to isolate single crystals of this compound so far.

The asymmetric unit of tribenzylammonium chloride consists of one third of a (C_6_H_5_CH_2_)_3_NH^+^ cation and an Cl^-^ anion (Fig. 1). The cationic molecule is completed by the symmetry operation associated with a threefold rotation axis. The N—C bond length within the cation [N–C1 1.5145 (17)] is nearly identical to that observed in tris(tribenzylammonium) hexachloridoplatinate(IV) chloride (Yousefi *et al.*, 2007), in tribenzylammonium l,l,l,l,2,2,2,3,3,3-decacarbonyl-2,3-*m*-hydrido-*2,3-m-*sulfonyl*-triangulo-*triosmium (Jarvinen *et al.*, 1988), or in dibenzylazanium (oxalato-*_k_*^2^*O*,*O*')triphenylstannate(IV) (Gueye *et al.*, 2012). The C–N–C angles [C1–N–C1^ii^ 111.16 (10)°] indicate a slight angular distortion in the tetrahedral environment.

In the crystal, the chloride anion is linked to the tribenzylammonium cation *via* N—H···Cl hydrogen bonding (Table 1). In addition and from a supramolecular point of view, the chloride anions are also in intermolecular weak interaction with three methylinic protons of the benzyl groups of neighboring cations (Table 1). The observed distances are in the range of those reported in literature for such interactions, for example in [(C_6_H_5_CH_2_Ph_3_P]^+^[SnPh_3_Cl_2_]^-^ (Traore *et al.*, 2013). The combination of N—H···Cl and C—H···Cl hydrogen bonding interactions leads to the formation of infinite chains along [001] (Fig. 2).

### 2. Experimental

All chemicals were purchased from Sigma-Aldrich and were used without further purification. Crystals of the title compound were obtained by reacting [(C_6_H_5_CH_2_)_3_NH]_2_[HPO_4_] (0.300 g, 0.446 mmol), previously synthesized from phosphoric acid (98%_wt_) and tribenzylamine, with Sn(CH_3_)_3_Cl (0.088 g, 0.446 mmol) in 15 ml of ethanol (98% purity). The mixture was stirred for around two hours at room temperature. Colorless crystals were obtained after one week by slow solvent evaporation.

### 3. Refinement

The H atoms,on carbon and nitrogen atoms were placed at calculated positions using a riding model with C—H = 0.95 Å (aromatic), or 0.99 Å (methylene) or N—H = 1.00 Å (amine) with *U*_iso_(H) = 1.2*U*_eq_(C or N). Intensity data revealed twinning by merohedry. The twin law was found by using TwinRotMat implemented in *PLATON* (Spek, 2009). The use of the twin law (-*h-k*, *k*, -*l*) and a refined twin component ratio of 0.93:0.07 reduced the reliability factor *R* (I>2*σ*(I)) from 0.042 to 0.021. The three reflections (-1 2 0; 1 1 0; -1 1 1) were affected by the beam stop and were omitted from the refinement.

### Crystal data

**Table:**

C_21_H_22_N^+^·Cl^−^	*D*_x_ = 1.174 Mg m^−^^3^
*M**_r_* = 323.85	Mo *K*α radiation, λ = 0.71073 Å
Trigonal, *R*3	Cell parameters from 2745 reflections
Hall symbol: R 3	θ = 1.0–27.5°
*a* = 15.3833 (8) Å	µ = 0.21 mm^−^^1^
*c* = 6.7051 (3) Å	*T* = 115 K
*V* = 1374.15 (18) Å^3^	Prism, colourless
*Z* = 3	0.47 × 0.27 × 0.12 mm
*F*(000) = 516	

### Data collection

**Table:**

Nonius KappaCCD diffractometer	1047 independent reflections
Radiation source: X-ray tube, Siemens KFF Mo 2K-180	1045 reflections with *I* > 2σ(*I*)
Graphite monochromator	*R*_int_ = 0.016
Detector resolution: 512 x 512 pixels mm^-1^	θ_max_ = 27.5°, θ_min_ = 3.4°
φ and ω scans	*h* = −18→17
Absorption correction: multi-scan (Blessing, 1995)	*k* = −19→10
*T*_min_ = 0.923, *T*_max_ = 0.963	*l* = −8→6
1884 measured reflections	

### Refinement

**Table:**

Refinement on *F*^2^	Hydrogen site location: inferred from neighbouring sites
Least-squares matrix: full	H-atom parameters constrained
*R*[*F*^2^ > 2σ(*F*^2^)] = 0.021	*w* = 1/[σ^2^(*F*_o_^2^) + 0.8302*P*] where *P* = (*F*_o_^2^ + 2*F*_c_^2^)/3
*wR*(*F*^2^) = 0.052	(Δ/σ)_max_ < 0.001
*S* = 1.10	Δρ_max_ = 0.12 e Å^−^^3^
1047 reflections	Δρ_min_ = −0.11 e Å^−^^3^
71 parameters	Absolute structure: Flack parameter determined using 348 quotients [(*I*^+^)-(*I*^-^)]/[(*I*^+^)+(*I*^-^)] (Parsons *et al.*, 2012)
1 restraint	Absolute structure parameter: 0.01 (4)
Primary atom site location: iterative	

### Special details

**Table:**

Geometry. All e.s.d.'s (except the e.s.d. in the dihedral angle between two l.s. planes) are estimated using the full covariance matrix. The cell e.s.d.'s are taken into account individually in the estimation of e.s.d.'s in distances, angles and torsion angles; correlations between e.s.d.'s in cell parameters are only used when they are defined by crystal symmetry. An approximate (isotropic) treatment of cell e.s.d.'s is used for estimating e.s.d.'s involving l.s. planes.
Refinement. Refined as a 2-component twin.

### Fractional atomic coordinates and isotropic or equivalent isotropic displacement parameters (Å^2^)

**Table:**

	*x*	*y*	*z*	*U*_iso_*/*U*_eq_	
Cl	0.6667	0.3333	0.80013 (9)	0.02215 (17)	
N	0.6667	0.3333	0.2481 (3)	0.0146 (5)	
H	0.6667	0.3333	0.0990	0.018*	
C1	0.56631 (12)	0.31837 (13)	0.3169 (3)	0.0168 (3)	
H1A	0.5122	0.2574	0.2520	0.020*	
H1B	0.5604	0.3064	0.4626	0.020*	
C2	0.55016 (13)	0.40504 (12)	0.2725 (3)	0.0176 (4)	
C3	0.51543 (14)	0.41462 (15)	0.0861 (3)	0.0231 (4)	
H3	0.5043	0.3676	−0.0165	0.028*	
C4	0.49715 (17)	0.49308 (17)	0.0510 (3)	0.0313 (5)	
H4	0.4731	0.4991	−0.0757	0.038*	
C5	0.51373 (16)	0.56246 (15)	0.1988 (4)	0.0332 (5)	
H5	0.5011	0.6159	0.1738	0.040*	
C6	0.54881 (15)	0.55355 (16)	0.3832 (4)	0.0313 (5)	
H6	0.5613	0.6016	0.4844	0.038*	
C7	0.56583 (14)	0.47482 (14)	0.4207 (3)	0.0234 (4)	
H7	0.5884	0.4683	0.5487	0.028*	

### Atomic displacement parameters (Å^2^)

**Table:**

	*U*^11^	*U*^22^	*U*^33^	*U*^12^	*U*^13^	*U*^23^
Cl	0.0273 (2)	0.0273 (2)	0.0118 (3)	0.01366 (12)	0.000	0.000
N	0.0156 (6)	0.0156 (6)	0.0128 (13)	0.0078 (3)	0.000	0.000
C1	0.0151 (7)	0.0189 (8)	0.0162 (8)	0.0084 (7)	0.0009 (7)	0.0013 (7)
C2	0.0139 (8)	0.0187 (8)	0.0202 (9)	0.0080 (7)	0.0013 (6)	−0.0002 (7)
C3	0.0240 (9)	0.0268 (9)	0.0220 (10)	0.0155 (8)	−0.0003 (7)	0.0000 (7)
C4	0.0317 (11)	0.0362 (11)	0.0349 (10)	0.0237 (9)	0.0028 (9)	0.0099 (10)
C5	0.0268 (10)	0.0240 (10)	0.0558 (15)	0.0178 (8)	0.0105 (10)	0.0066 (10)
C6	0.0227 (9)	0.0242 (9)	0.0478 (14)	0.0123 (8)	0.0087 (9)	−0.0072 (9)
C7	0.0186 (9)	0.0258 (10)	0.0254 (10)	0.0108 (8)	0.0014 (7)	−0.0053 (8)

### Geometric parameters (Å, º)

**Table:**

N—H	1.0000	C3—H3	0.9500
N—C1^i^	1.5145 (17)	C3—C4	1.389 (3)
N—C1^ii^	1.5145 (17)	C4—H4	0.9500
N—C1	1.5145 (17)	C4—C5	1.384 (3)
C1—H1A	0.9900	C5—H5	0.9500
C1—H1B	0.9900	C5—C6	1.383 (3)
C1—C2	1.503 (2)	C6—H6	0.9500
C2—C3	1.396 (3)	C6—C7	1.384 (3)
C2—C7	1.392 (2)	C7—H7	0.9500
			
C1^ii^—N—H	107.7	C2—C3—H3	120.1
C1^i^—N—H	107.7	C4—C3—C2	119.86 (19)
C1—N—H	107.7	C4—C3—H3	120.1
C1^ii^—N—C1^i^	111.16 (10)	C3—C4—H4	119.7
C1^i^—N—C1	111.16 (10)	C5—C4—C3	120.6 (2)
C1^ii^—N—C1	111.16 (10)	C5—C4—H4	119.7
N—C1—H1A	108.7	C4—C5—H5	120.2
N—C1—H1B	108.7	C6—C5—C4	119.63 (19)
H1A—C1—H1B	107.6	C6—C5—H5	120.2
C2—C1—N	114.39 (13)	C5—C6—H6	119.9
C2—C1—H1A	108.7	C5—C6—C7	120.2 (2)
C2—C1—H1B	108.7	C7—C6—H6	119.9
C3—C2—C1	120.83 (16)	C2—C7—H7	119.7
C7—C2—C1	120.07 (16)	C6—C7—C2	120.62 (19)
C7—C2—C3	119.04 (17)	C6—C7—H7	119.7
			
N—C1—C2—C3	83.7 (2)	C2—C3—C4—C5	0.5 (3)
N—C1—C2—C7	−99.0 (2)	C3—C2—C7—C6	−1.0 (3)
C1^ii^—N—C1—C2	174.09 (11)	C3—C4—C5—C6	0.0 (3)
C1^i^—N—C1—C2	49.7 (2)	C4—C5—C6—C7	−1.0 (3)
C1—C2—C3—C4	177.39 (18)	C5—C6—C7—C2	1.5 (3)
C1—C2—C7—C6	−178.38 (17)	C7—C2—C3—C4	0.1 (3)

Symmetry codes: (i) −*x*+*y*+1, −*x*+1, *z*; (ii) −*y*+1, *x*−*y*, *z*.

### Hydrogen-bond geometry (Å, º)

**Table:**

*D*—H···*A*	*D*—H	H···*A*	*D*···*A*	*D*—H···*A*
N—H···Cl^iii^	1.00	2.00	3.004 (2)	180
C1—H1*B*···Cl	0.99	2.70	3.5470 (18)	144
C3—H3···Cl^iii^	0.95	3.06	3.683 (2)	125

Symmetry code: (iii) *x*, *y*, *z*−1.
